# Curcumin as a Therapeutic Strategy in Liver Diseases

**DOI:** 10.3390/nu11102498

**Published:** 2019-10-17

**Authors:** Rita Rezzani, Caterina Franco, Luigi Fabrizio Rodella

**Affiliations:** 1 Anatomy and Physiopathology Division, Department of Clinical and Experimental Sciences, University of Brescia, 25123 Brescia, Italy; c.franco@studenti.unibs.it (C.F.); luigi.rodella@unibs.it (L.F.R.); 2 Interdipartimental University Center of Research “Adaption and Regeneration of Tissues and Organs-(ARTO)”, University of Brescia, 25123 Brescia, Italy

**Keywords:** acute and chronic liver disease, oxidative stress, natural antioxidants, curcumin

## Abstract

Liver diseases are classified as acute and chronic hepatic failures. In particular, chronic pathologies are the most common diseases in the World. Chronic pathologies of liver disease are the most common diseases in the world. There are many causes that induce a progressive and irreversible degeneration of the hepatic parenchyma, but, in general, they lead to the destruction of the normal balance between reactive oxygen stress (ROS) formation and ROS degradation within the liver. The prevalence of disabling diseases, including the hepatic diseases, is increasingly widespread, and it is important to find a safe, inexpensive, accessible and effective way to face this condition. Many recent studies have focused on different natural antioxidants, which could restore the physiological hepatic environment, thereby allowing the normal functioning of this organ. Natural products have been used to discover new leads for treating several diseases; among them, it is important to emphasize curcumin, which is a polyphenol obtained from *Curcuma longa* Linn, a plant naturally found throughout tropical and subtropical regions of the world.

## 1. Editorial

To our knowledge, liver diseases are the most common diseases in the world, affecting more than 10% of the population [1]. They account for approximately two million deaths worldwide every year, representing 3.5% of all deaths. Moreover, it is not possible to have accurate and recent statistics because the underlying causes of liver damage, the trigger points for its malfunction, are several and sparsely identified in the different regions around the world [2]. The WHO (World Health Organization) reported the clinical data describing the incidence of hepatic diseases and liver-related mortality [3]. These findings are summarized in Table 1; in particular, cirrhosis is the 11th most common cause of death, while liver cancer is the 16th most common cause [4].

Further, the WHO has provided many researches and statistics evaluating not only the mortality associated with liver damages, but also the morbidity. Table 2 reports these findings as disease-related disability-adjusted life years (DALYs) and years of life lost (YLLs) [5].

**Figure 1:**
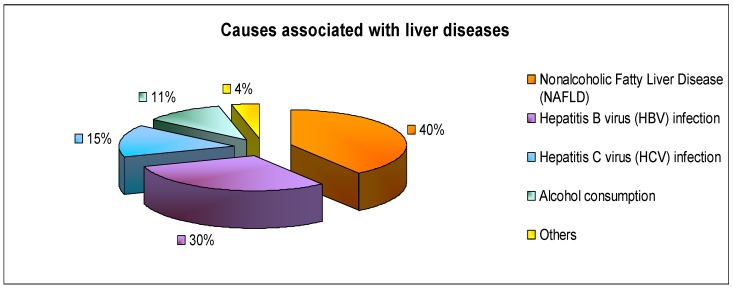
A graphical schematic representation of the main causes associated with liver damages.

Therefore, it is not surprising that the liver is considered one of the most important organs for good health. It is involved in the performance of many functions and reacts particularly sensitively to different stimuli [1]. Recently, it has been demonstrated that nonalcoholic fatty liver disease (NAFLD) is a predominant cause (40% of cases) of liver dysfunction. The second most frequent cause are hepatitis B virus (HBV) infections (30%). Hepatitis C virus (HCV) infections (15%) and alcohol consumption (11%) are the other common causes of hepatic dysfunctions (Figure 1) [6].

Moreover, the constant exposition of the liver to viruses, pathogens and toxins, together with injury associated with overnutrition, obesity and metabolic syndrome, lead to the development of liver abnormalities. Normally, these alterations are related to the lipid accumulation in hepatocytes, which starts with simple steatosis and ends with cirrhosis and hepatocellular carcinoma.

## 2. Liver in Acute and Chronic Diseases

In this context, it is possible to describe two main forms of liver diseases; in particular, the first one, an acute, usually irreversible damage, and the second one, characterized by a chronic, less clear manifestation [7]. 

Acute liver failure (ALF) is a rare disease that could affect many organs, leading to a systemic syndrome. It is a condition that can arise in a patient without any pre-existing liver damages in less than 26 weeks [8]. If it is quickly diagnosed, it is reversible and thus it is essential to clearly indicate the cause of liver dysfunction in order to define an effective therapy. The causes of the failure can be due to drugs (especially, paracetamol), hepatitis viruses A and B, pregnancy and other sources that rapidly determine the necrosis of liver tissues [7]. It is important to stress that it is fundamental to identify each different cause of hepatic injury to define a specific relevant therapeutic intervention.

On the other side, chronic liver failure (CLF) is characterized by chronic progressive damage to liver cells that slowly leads to a loss of liver function, which is clinically manifested only when 70% of the hepatic parenchyma is compromised.

While ALF could be reversed through liver regeneration, CLF is usually irreversible [7]. There are many mechanisms associated with persistent damage to liver cells, but overall, they define an increase in oxidative stress. The production of ROS is part of the aerobic life and of the physiological cellular metabolism, but continuous exposure to chronic stress progressively leads to structural changes with the oxidation of substrates such as lipids, proteins and DNA, definitively compromising cellular functionality [9].

**Figure 2:**
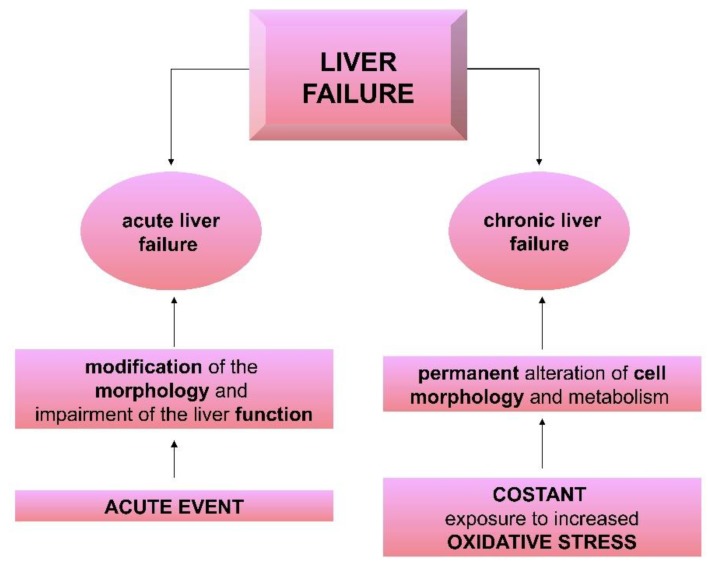
Schematic classification of liver failure, underlining the different mechanisms behind the evolution of the disease.

The hepatic diseases and the possible mechanisms of the hepatic injures are reported in Figure 2.

## 3. Oxidative Stress as Major Causes of Liver Diseases

A delicate balance is important for maintaining the redox physiological homoeostasis that characterizes the healthy liver. An increased generation of free radicals, together with a decreased antioxidant defences in hepatocytes, promotes the progression of oxidative stress, leading to an alteration of the complex oxidant and antioxidant system that defines one of the main causes of liver dysfunctions [9]. Thus, oxidative stress is implicated in several forms of livers diseases (i.e., viral hepatitis, necrosis, fibrosis, cirrhosis and hepatocellular carcinoma) and leads to several damages of hepatocytes, including proteins, lipids and DNA degradation [6]. It has been proposed that the release of ROS from injured hepatocytes causes the activation of several type of other cells leading to their proliferation and invasiveness, resulting in an inflammatory response, collagen production and the initiation of fibrosis [10].

**Figure 3:**
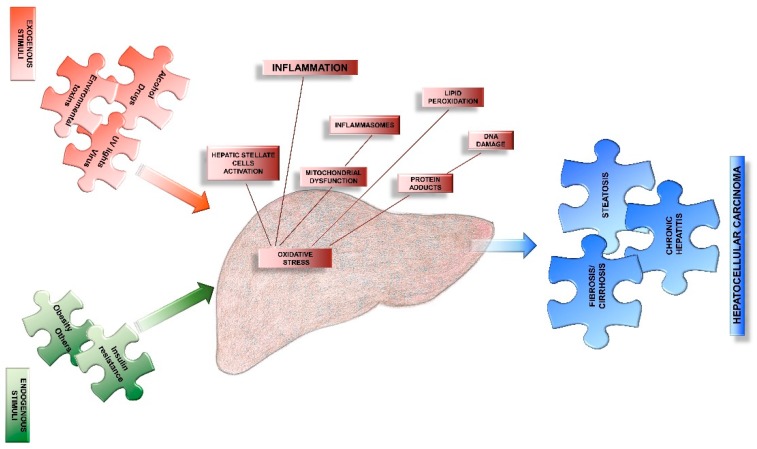
A schematic summary of the main determinants that are responsible to induce an increased intrahepatic oxidative stress. This summary underlines the pathways that are activated by endogenous and exogenous stimuli and the terminal changes involving hepatic parenchyma.

In the end, these changes modify many signal pathways in the hepatocytes, also inducing other alterations in physiological activities of the liver (Figure 3).

Above all, the increase of ROS hepatic content represents a trigger point in the development of numerous chronic diseases, as previously reported. Moreover, oxidative stress is a phenomenon that should be better investigated not only for its role in the pathogenesis of these pathological alterations, but also as a marker useful in the diagnosis and definition of the degree of liver damage. In the end, the evaluation of ALF or CLF is important for evaluating the prognosis and response to treatment [9,11].

## 4. Curcumin and Oxidative Stress Associated to the Liver Injuries

The high incidence of liver diseases induced clinicians and scientists to find a possible safe, inexpensive and effective intervention. During the last few years, many researchers have increasingly addressed and deepened their studies around substances of natural origin. In fact, natural products have been used to discover new leads for treating several diseases [12]. Among them, it is important to emphasize curcumin, which is a polyphenol obtained from *Curcuma longa* Linn, a plant naturally found throughout tropical and subtropical regions of the world [13]. It is known as a strong antioxidant and anti-inflammatory agent with different pharmacological effects; the efficacy and safety of this polyphenol have been shown against several human diseases including cancer, diabetes, and inflammatory conditions [14]. Moreover, it has been demonstrated that curcumin’s related compounds have been associated with the inhibition of radical formation and, consequently, the reduction of DNA damage [15].

Chemically, curcumin is a compound characterized by the presence of a variety of antioxidant groups, and is considered a very strong lipid-soluble antioxidant [16]. Experimental studies have shown that curcumin is able to determine a significative reduction of ductal proliferation and portal inflammation. These macroscopical restorations are also supported by an effective reduction of glutathione, nitric oxide and tumor necrosis factor-α levels [17]. Furthermore, the hepatoprotective activity of this antioxidant is associated with an inhibition of lipid peroxidation and an enhancement in the levels of antioxidant enzymes (i.e., thiols, superoxide dismutase and catalase) as reported by Ghoreshi et al. [18]. These findings suggest a potential role of curcumin against inflammatory and reperfusion-related injuries.

The liver is an important organ actively involved in many metabolic functions; it is a frequent target for a number of toxicants, and several researchers have underlined the protective capacity of curcumin against liver injury associated to some xenobiotics. The protective effects are linked to the ability of curcumin to induce the Nrf2/antioxidant response elements/Kelch-like family member 19 (Nrf2/ARE/Keap1) pathway [19]. Other authors suggest that curcumin shows a significant reduction of the levels of inducible nitric oxide and nuclear factor-κB (NF-κB) in acute thioacetamide hepatotoxicity rats [20]. It is known that the inhibition of NF-κB translocation in the nucleus and the decreasing in its DNA binding activity leads to a reduction in the production of the proinflammatory cytokines, improving the inflammatory state that is the main cause of liver injures [21].

It is important to report that many findings suggest the efficacy of curcumin’s supplementation during liver fibrosis and cirrhosis. In these conditions, in fact, it is possible to identify a higher deposition of extracellular matrix, where collagen represents one of the most abundant proteins [22]. The transforming growth factor β and other proteins mediate the excessive accumulation of collagen that represents the starting point for the development of cirrhosis.

Generally, several studies associated with these chronic conditions showed an important improvement with the supplementation of curcumin, characterized by a significant reduction in serum and tissue cholesterol profiles, glutamate pyruvate transaminase, glutamic oxaloacetic transaminase and alkaline phosphatase [23]. In addition to the most obvious effects confirmed by changes in serum values of liver enzymes and modulation of the main markers of inflammation, less common, but increasingly frequent, studies focus on genetic and epigenetic changes associated with the administration of curcumin. To our knowledge, little is known around this point, but it is clear that there is a link between oxidative stress, epigenetics and NAFLD through the mitochondria. DNA methylation and histone modifications have been considered for their implication in the development of liver [24].

## 5. Conclusions

Today, liver diseases and, in particular, chronic hepatic diseases associated with oxidative stress are being more and more studied. Recent researches have focused on new therapeutic approaches that are very important.

To date, many preclinical studies have been carried out around the effectiveness of dietary curcumin as an instrument in the management of oxidative-associated liver diseases. However, there are few studies showing the value of curcumin in the prevention and treatment of hepatic diseases in humans.

In this context, this editorial and Farzaei et al.’s [6] review underline that further in vivo and vitro studies are very important to identify the exact bioavailability, bioefficacy and cellular pathways on which curcumin is involved in a possible therapeutic regime.

## Figures and Tables

**Table 1:** The whole number of deaths and the corresponding percentage of the total number of deaths for each Country are reported. The WHO reports, in the Global Health Estimates (Geneva, 2016), the number of liver-related deaths by cause, age, sex, Country and Region during the years 2000–2015 [3].

	Deaths (000s)	% of Total Deaths
World	1162	2.1
East Asia and Pacific	328	2.0
Europe and Central Asia	115	1.2
Latin America and Caribbean	98	2.7
Middle East and North Africa	77	3.5
North America	50	1.7
South Asia	314	2.5
Sub-Saharan Africa	179	1.9

**Table 2:** Disease-related disability-adjusted life years (DALY) and of years of life lost (YLL) due to liver-related diseases according to the WHO. By region, collected during the years 2000–2015 [5].

	DALY (000s)	YLL (000s)
Global	41,486	40,986
WHO African Region	7242	7195
WHO Region of the Americas	4890	4826
WHO South-East Asia Region	15,581	15,450
WHO European Region	33,608	3502
WHO Eastern Mediterranean Region	3409	3371
WHO Western Pacific Region	6518	6407
